# Adaptation of pain scales for parent observation: are pain scales and symptoms useful in detecting pain of young children with the suspicion of acute otitis media?

**DOI:** 10.1186/s12887-018-1361-y

**Published:** 2018-12-20

**Authors:** Johanna M. Uitti, Sanna Salanterä, Miia K. Laine, Paula A. Tähtinen, Aino Ruohola

**Affiliations:** 1 Department of Paediatrics and Adolescent Medicine, Turku University Hospital, Turku, Finland; 2 Department of Paediatrics and Adolescent Medicine, University of Turku, Turku, Finland; 3 Department of Nursing Science, University of Turku, Turku, Finland; 4 Hospital District of Southwest Finland, Turku, Finland; 5 Department of Clinical Microbiology, Turku University Hospital, Turku, Finland

**Keywords:** Child, Otitis media, Pain scales, Parents, Respiratory tract infection

## Abstract

**Background:**

The assessment of ear pain is challenging in young, mostly preverbal children. Our aim was to investigate whether pain scales are useful tools for parents to detect pain in their young children with the suspicion of acute otitis media (AOM), and to assess associations between 16 symptoms and the severity of pain.

**Methods:**

This cross-sectional study included 426 children (6–35 months) with symptoms suggestive of AOM. We surveyed symptoms and pain via parental interview. As part of the interview, parents assessed their child’s pain by using two pain scales: The Faces Pain Scale-Revised (FPS-R) and the Face, Legs, Activity, Cry, Consolability (FLACC) Scale. The outcome of interest was moderate/severe pain. We used the χ^2^ test or Fisher’s test as applicable to compare the severity of pain between three parental pain assessment methods (the parental interview, the FPS-R and the FLACC Scale). We also used multivariable logistic regression models to study the association between the severity of pain and AOM and to study the association between symptoms and the severity of pain.

**Results:**

In children with AOM (*n* = 201), pain was assessed by parents as moderate/severe in 65% via interview; 90% with the FPS-R; and 91% with the FLACC Scale (*P* < 0.001). In children without AOM (*n* = 225), the percentages were 56, 83 and 88%, respectively (*P* < 0.001). Between children with and without AOM, the occurrence of moderate/severe pain did not differ with any of the pain evaluation methods. Of symptoms, ear pain reported by child and restless sleep were significantly associated with moderate/severe pain, regardless of the pain evaluation method.

**Conclusions:**

It seems that nearly all the children with respiratory tract infection, either with or without AOM, might suffer from moderate/severe pain. Without pain scales, parents may underestimate their child’s pain. Of symptoms, ear pain reported by child and restless sleep might indicate pain in children with respiratory tract infection. We suggest that the adaptation of pain scales for parent observation is a possibility in children with respiratory tract infection which, however, requires further studies.

**Trial registration:**

www.clinicaltrials.gov, identifier NCT00299455. Date of registration: March 3, 2006.

**Electronic supplementary material:**

The online version of this article (10.1186/s12887-018-1361-y) contains supplementary material, which is available to authorized users.

## Background

Acute otitis media (AOM) is one of the most common diseases in early childhood, causing variety of symptoms. Ear pain is considered as the most important and specific symptom of AOM and parents perceive it as one of the greatest burden in young children with AOM [1]. Furthermore, ear pain is used as one of the key criteria when defining the severity of AOM, which, in turn, guides the management of AOM [2]. Consequently, it is crucial to be able to reliably assess whether young children have any ear pain.

The assessment of ear pain is challenging in young, mostly preverbal children. First, they cannot provide self-reports which are often considered a primary source for estimates of pain intensity [3]. Therefore, the pain assessment is based on the opinion of parents and health care professionals. Ear pain of preverbal children is suggested to emerge as various non-specific symptoms, according to The American Academy of Pediatrics AOM guideline [2]. Nevertheless, the guideline does not give any instructions how to further convert the non-specific symptoms as mild, moderate or severe pain behavior. The study of Shaikh et al. [4] suggested ear rubbing and fussiness to be the most important symptoms in influencing parental perception of ear pain in preverbal children with AOM. However, as they stated, their results are preliminary and are based on hypothetical patient scenarios. To our knowledge, the adaptation of pain scales for parental use to assess acute, non-procedural pain in young children in an outpatient setting has not been investigated.

We adapted two well-established pain scales for parent observation. Our primary aim was to investigate whether pain scales are useful tools for parents to detect pain in their young, mostly preverbal children with the parental suspicion of AOM. Furthermore, we investigated which symptoms are associated with moderate/severe pain in young children.

## Methods

### Study population

This study was part of a project examining diagnostics and treatment of AOM at the primary care level (www.clinicaltrials.gov, identifier NCT00299455) between 2006 and 2008 in Turku, Finland [5]. Written informed consent was obtained from a parent of all children before they could participate in the study. All visits were free of charge, and no compensation for participation was given. The study protocol was approved by The Ethics Committee of the Hospital District of Southwest Finland (reference number: Dnro 4/2016).

Children 6 to 35 months of age were eligible when they had acute symptoms and parental suspicion of AOM. The exclusion criteria have been previously described in detail [5]. The focus of the study is on the child’s symptoms and clinical findings at the time of an enrolment, on day 1. In this cross-sectional study, we used the same cohorts as in our previous reports regarding symptoms, nasopharyngeal bacteria, and respiratory viruses [6–8].

### Symptom questionnaire

Before examining the child the study physician interviewed the parents about the occurrence of 17 symptoms of their child by using a standardized, structured symptom questionnaire, which is described in detail below. We defined fever as temperature ≥ 38 °C within the preceding 24 h, but we also accepted if parents reported that their child had been febrile even though temperature had not been measured with a thermometer. We asked about three *ear-related symptoms*: parentally reported ear pain, ear pain reported by child (the child verbally expressed of having ear pain), and ear rubbing. Parents also assessed the severity of their child’s ear pain classified as mild, moderate or severe. Furthermore, we asked parents to assess their child’s pain with the pain scales (described in detail below). Apart from this, we interviewed the parents about *non-specific symptoms*: irritability, excessive crying, restless sleep, decreased activity, poor appetite; *respiratory symptoms*: rhinitis, nasal congestion, cough, hoarse voice, conjunctivitis, mucus vomiting; and *gastrointestinal symptoms*: vomiting, and diarrhea. Finally, the study physician asked about the duration of the parental suspicion of AOM.

### Pain scales

**Fig. 1:**
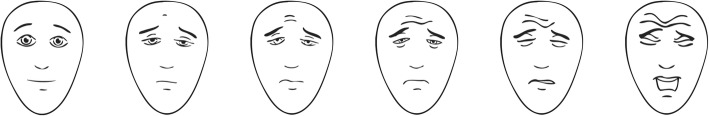
“Faces Pain Scale - Revised (FPS-R)”. www.iasp-pain.org/fpsr. Copyright ©2001, International Association for the Study of Pain®. Reproduced with permission

Due to the shortage of validated pain scales to obtain parent measures of acute and nonsurgical pain of their young child, we performed a preliminary study and used two pain scales and adapted them for parent observation in children with the suspicion of AOM. First, we used The Faces Pain Scale-Revised (FPS-R) (Fig. 1) [9], which is a validated self-report tool for children measuring the pain intensity, but it has likewise been previously adapted for parental use as an observational pain measurement tool [10, 11]. The FPS-R consists of 6 horizontally positioned faces, representing increasing levels of pain from left (“no pain”) to right (“very much pain”), scored as 0–2–4-6-8-10 [9]. The parents pointed out the face which best reflected their child’s pain at its worst within the preceding 24 h. The FPS-R was chosen since it is easy to comprehend and does not require a lot of time or special skills [12, 13]. However, FPS-R is not a behaviorally anchored rating scale [14] and hence another scale was added to the study. Second, we used The Face, Legs, Activity, Cry, Consolability (FLACC) Scale, which is an observational pain measurement tool (Table 1) [15]. The FLACC Scale includes 5 behavioral categories: facial expression, leg movement, bodily activity, cry or verbalization, and consolability. The parents rated their child’s pain at its worst within the preceding 24 h in each category on a scale of 0 to 2, thus an overall pain score ranging from 0 to 10. The FLACC Scale has previously been translated into Finnish. The FLACC Scale was chosen since it is a well-established and validated tool, suitable for children from 0 to 18 years of age [14, 16]. Furthermore, the FLACC Scale has low burden, it has excellent inter-rater reliability, and moderate concurrent validity and it is recommended for evaluating pain in brief painful events [14].

**Table 1:** The FLACC scale. Each of the five categories Face; Legs; Activity; Cry; Consolability is scored from 0 to 2, which results in a total score between 0 and 10

Categories	Scoring		
	0	1	2
Face	No particular expression or smile	Occasional grimace or frown, withdrawn, disinterested	Frequent to constant quivering chin, clenched jaw
Legs	Normal position or relaxed	Uneasy, restless, tense	Kicking, or legs drawn up
Activity	Lying quietly, normal position, moves easily	Squirming, shifting back and forth, tense	Arched, rigid or jerking
Cry	No cry (awake or asleep)	Moans or whimpers; occasional complaint	Crying steadily, screams or sobs, frequent complaints
Consolability	Content, relaxed	Reassured by occasional touching, hugging or being talked to, distractable	Difficult to console or comfort

From Merkel SI, Voepel-Lewis T, Shayevitz JR, Malviya S. The FLACC: A behavioral scale for scoring postoperative pain in young children. Pediatr Nurs. 1997;23:293–297 [15]

We had three conventionally used clinical pain categories: with the FPS-R, the scores 0 and 2 were classified as “none or mild”, 4 and 6 as “moderate” and 8 and 10 as “severe” pain. With the FLACC scale, the scores from 0 to 3 were classified as “none or mild”, from 4 to 6 as “moderate” and from 7 to 10 as “severe” pain or discomfort, respectively [12, 17–19].

After the symptom questionnaire, the study physician performed clinical examination on the child, including tympanometry, pneumatic otoscopy, and video otoscopy, as described in detail elsewhere [5]. The diagnosis of AOM was based on the following three criteria. First, middle ear effusion had to be detected by pneumatic otoscopy (at least two of the following signs on tympanic membrane: bulging position, decreased or absent mobility, abnormal color or opacity not due to scarring, or air-fluid interfaces). Second, at least one acute inflammatory sign of tympanic membrane had to be identified (distinct erythematous patches/streaks, or increased vascularity over full/bulging/yellow convexity). Third, there had to be symptoms and signs of acute infection.

### Statistical analysis

The outcome of interest was moderate/severe pain. We compared the proportions with χ^2^ test or Fisher’s test as applicable. We compared the medians with the Mann-Whitney *U* test. Absolute percentage-point differences in rates and 95% confidence intervals (CI) were calculated. We used multivariable logistic regression models for two purposes: first, to study the association between the severity of pain and AOM. We calculated the odds ratios (ORs) with 95% confidence intervals (CI) for AOM and adjusted the models by age (1 month as a unit), use of analgesics (yes vs. no) and the duration of the parental suspicion of AOM (1 h as a unit); and second, to study the association between symptoms and the severity of pain. We calculated the ORs (with 95% CI) for moderate/severe pain and adjusted the models by age (1 month as a unit), diagnosis of AOM (yes vs. no), and use of analgesics (yes vs. no). We performed statistical analyses by using SPSS version 22.0 (IBM SPSS Statistics, IBM Corporation, Armonk, NY).

## Results

Children were enrolled to the study between March 16, 2006 and December 5, 2008, excluding June and July of each year. The study population consisted of 426 children (6–35 months). Of those, 201 (47%) had AOM (AOM group) and 225 (53%) did not have AOM (non-AOM group). The patient characteristics are shown in Table 2. Children with AOM had used analgesics more often than children without AOM (60% [121/201] vs. 48% [109/225], *P* = 0.02).

**Table 2:** Characteristics of 426 children with and without acute otitis media

	AOM (*N* = 201)	Non-AOM (*N* = 225)	*P*
Median (range) age, mo	15 (6–35)	13 (6–35)	0.37
Age, n (%)			0.75
6–11 mo	76 (38)	91 (40)	
12–23 mo	85 (42)	95 (42)	
24–35 mo	40 (20)	39 (17)	
Male gender, n (%)	109 (54)	118 (52)	0.71
Symptom interview answered by			0.25
Mother, n (%)	144 (72)	174 (77)	
Father, n (%)	22 (11)	24 (11)	
Both parents, n (%)	33 (16)	27 (12)	
Other guardian, n (%)	2 (1)	0 (0)	
Median (Q1, Q3)^a^ duration (h) of the parental suspicion of AOM	21 (12, 38)	24 (16, 48)	0.04
The use of analgesics ≤24 h, n (%)	121 (60)	109 (48)	0.02
Number of previous AOM episodes, n (%)^b^			0.25
0 episodes, n (%)	62 (31)	70 (32)	
1–3 episodes, n (%)	108 (54)	105 (47)	
> 3 episodes, n (%)	31 (15)	47 (21)	
Median (range) age at first AOM episode, mo^c^	9 (0–27)	9 (0–29)	0.47

^a^Q1, the 25th quartile; Q3, the 75th quartile

^b^Data were missing in 3/225 children without AOM

^c^Among those who had had at least one episode of AOM. Data were missing in 8/139 and 18/152 children with AOM and non-AOM

**Fig. 2:**
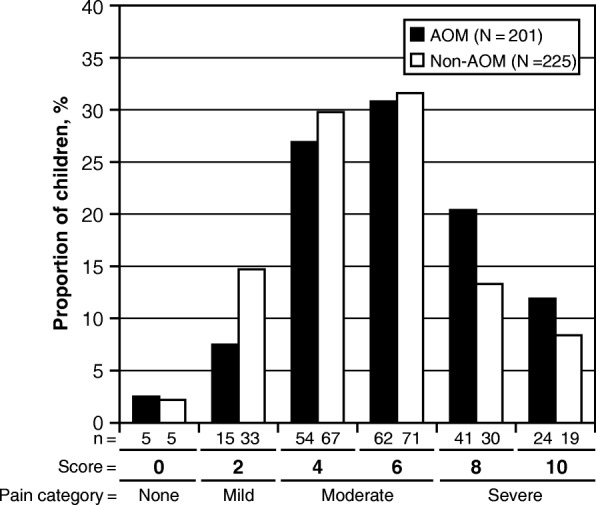
Distribution of the Faces Pain Scale-Revised (FPS-R) scores in children in the AOM group and in children in the non-AOM group. The numbers below the bars show the number of children with the score, indicating the numerator (n)

**Fig. 3:**
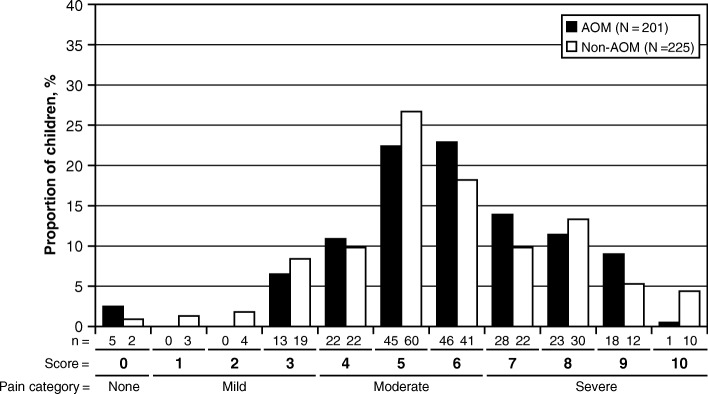
Distribution of the Face, Legs, Activity, Cry, Consolability (FLACC) Scale scores in children in the AOM group and in children in the non-AOM group. The numbers below the bars show the number of children with the score, indicating the numerator (n)

The distributions of the FPS-R and FLACC Scale scores in the AOM group and in the non-AOM group are presented in Fig. 2 and Fig. 3.

### Severity of pain in the AOM group and in the non-AOM group

**Fig. 4:**
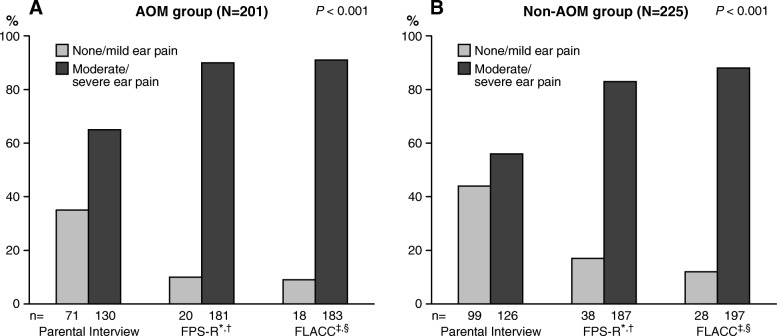
**a**, **b** The occurrence of none/mild and moderate/severe pain in the AOM group (**a**) and in the non-AOM group (**b**), assessed by parents via interview, with the Faces Pain Scale-Revised (FPS-R) and with the Face, Legs, Activity, Cry, Consolability (FLACC) Scale. Footnote: ^*^ Scores 0 and 2 were classified as none/mild pain, and scores 4, 6, 8 and 10 as moderate/severe pain, respectively. ^†^*P* < 0.001 for the comparison between none/mild pain and moderate/severe pain, assessed by parents with the FPS-R and via interview. ^‡^ Scores from 0 to 3 were classified as none/mild pain, and scores from 4 to 10 as moderate/severe pain, respectively. ^§^ *P* < 0.001 for the comparison between none/mild pain and moderate/severe pain, assessed by parents with the FLACC Scale and via interview

Figure 4a and Fig. 4b show the occurrence of none/mild and moderate/severe pain in the AOM group and in the non-AOM group, respectively. In the AOM group (Fig. 4a), parents assessed their child’s pain significantly more often as moderate/severe with the FPS-R and with the FLACC Scale, compared with the parental interview (*P* < 0.001). The rate difference for moderate/severe pain between the FPS-R and parental interview was 25% (95% CI, 17 to 34%), and between the FLACC Scale and parental interview 26% (95% CI, 18 to 35%), respectively.

In the non-AOM group (Fig. 4b), parents likewise assessed their child’s pain significantly more often as moderate /severe with the FPS-R and with the FLACC Scale, compared with the parental interview (*P* < 0.001). The rate difference for moderate/severe pain between the FPS-R and parental interview was 27% (95% CI, 19 to 36%), and between the FLACC Scale and parental interview 32% (95% CI, 23 to 40%), respectively.

### Comparison of pain between AOM group and non-AOM group

First, via parental interview, moderate/severe pain was reported in 130/201 (65%) children in the AOM group, compared with 126/225 (56%) children in the non-AOM group (*P* = 0.07). When parents had assessed their child to have moderate/severe pain via parental interview, the adjusted OR for AOM was 1.32 (95% CI, 0.88–1.98). Second, the parental pain assessment with the FPS-R showed moderate/severe pain in 181/201 (90%) children in the AOM group, compared with 187/225 (83%) children in the non-AOM group (*P* = 0.04). When parents had assessed their child to have moderate/severe pain with the FPS-R, the adjusted OR for AOM was 1.75 (95% CI, 0.97–3.15). Third, the parental pain assessment with the FLACC Scale showed moderate/severe pain in 183/201 (91%) children in the AOM group, compared with 197/225 (88%) children in the non-AOM group (*P* = 0.25). When parents had assessed their child to have moderate/severe pain with the FLACC Scale, the adjusted OR for AOM was 1.46 (95% CI, 0.77–2.75).

### Association of individual symptoms with moderate/severe pain

**Fig. 5:**
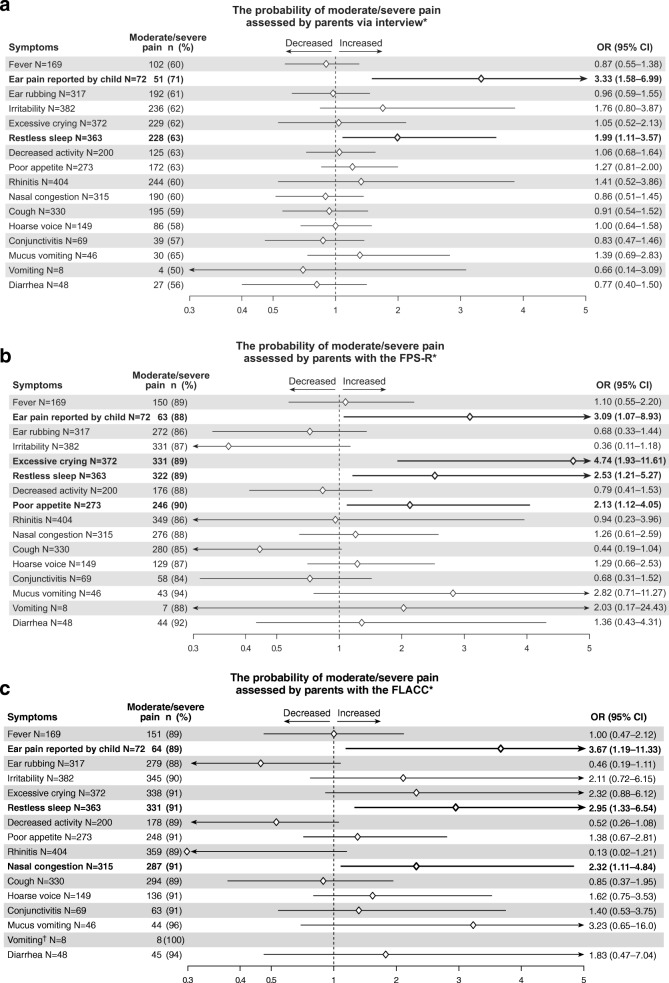
The occurrence and the probability of moderate/severe pain, assessed by parents via interview (**a**), with the Faces Pain Scale-Revised (FPS-R) (**b**) and with the Face, Legs, Activity, Cry, Consolability (FLACC) Scale (**c**), in relation to the presence of 15 parentally reported symptoms and ear pain reported by children in 426 children with the suspicion of AOM, analysed with multivariable logistic regression model and adjusted for age, diagnosis of AOM and use of analgesics. Footnote: ^*^ Diamonds indicate odds ratio (OR), lines 95% confidence intervals (CI), arrows are added when CI is beyond the scale. ^†^The association of moderate/severe pain with the symptom was 100%

Among all the 426 children with the parental suspicion of AOM, the associations between individual symptoms and moderate/severe pain are presented in Fig. 5a, b and c. As parents assessed their child’s pain via interview (Fig. 5a), ear pain reported by child and restless sleep had significant associations with moderate/severe pain. As parents assessed their child’s pain with the FPS-R (Fig. 5b), following symptoms had significant associations with moderate/severe pain: ear pain reported by child, excessive crying, restless sleep and poor appetite. Finally, as parents assessed their child’s pain with the FLACC Scale (Fig. 5c), following symptoms had significant associations with moderate/severe pain: ear pain reported by child, restless sleep and nasal congestion.

## Discussion

Our main finding is that pain scales, namely the FPS-R and the FLACC Scale, might be useful for detecting pain by parents in young children with respiratory tract infection (RTI), either with or without AOM. What is more, without the pain scales, parents may underestimate pain in young children with RTI. Furthermore, nearly all children might suffer from moderate/severe pain or distress during RTI, regardless of the diagnosis of AOM.

Parental pain assessment with the FPS-R and the FLACC Scale indicated that the great majority of the children with RTI, either with or without AOM, seem to suffer from moderate/severe pain. In contrast, when parents were being interviewed about their child’s pain, moderate/severe pain was reported only in two thirds of the children with RTI. The difference in the results between the pain assessment methods is obvious and thus requires further attention. Since the pain results cannot be compared with children’s self-reports of pain, the most reliable pain evaluation method cannot be stated for absolute certainty. It can be debated that in our study, children were conventionally classified as having moderate pain with the FPS-R scores of 4 or 6. On the contrary, the study of Tsze et al. proposes that only children with the FPS-R scores of 6 would be classified as having moderate pain, although considerable overlap of scores associated with mild and moderate pain could be seen in their study [20]. In our study, however, moderate/severe pain was detected at the similar rate with both the FPS-R and with the FLACC Scale, thus suggesting the reliability of our pain category classification for moderate pain with the FPS-R.

Worth noting, the FPS-R was originally designed and validated to be a self-report measure to assess the intensity of children’s acute pain from age 4 or 5 onward [9], and it is not validated for the observational use, although faces scales have also been adapted for global observational ratings by parents and nurses [10, 11, 14, 21]. On the contrary, the FLACC Scale, which was initially developed for evaluating postoperative pain in young children [15], has further been established as a valid observational measure for all kinds of pain in preverbal children by nurses [22], although its clinical utility has recently been challenged [23]. Thus, it should be acknowledged that neither of the pain scales are validated to assess acute, non-surgical pain of young children by parents. Therefore, we can only present preliminary results. For instance, parents may overestimate their child’s pain with faces scales and with the FLACC Scale [24, 25] although underestimation with the faces scale, as well as with the parental interview have likewise been reported [11, 26]. However, parents are considered as most reliable proxy for assessing young children’s possible pain, if the self-report is not possible, because children are often more expressive in the presence of parents than strangers, such as health care professionals [27]. Parents are likewise familiar with the child’s normal behavior and thus they are more able to discriminate child’s pain behavior from other aberrant behavior [14, 28].

Overall, there seems to be relatively pervasive and systematic tendency for proxy judgments to underestimate the pain experience of others [29]. However, direct observations of pain behavior and self-reports of pain intensity are more likely to be significantly related to each other, if the individual being studied has acute pain, instead of chronic pain, because nociception plays a greater role in the display of observable behavior among persons with acute pain [30]. In fact, acute pain of young children has recently been shown to be reliably assessed with the FLACC Scale by nurses [31]. Taken these findings together, we cautiously suggest that the FLACC Scale might also be used by parents in children with RTI. Since the pain results of the FLACC Scale and the FPS-R were highly similar, this implies that the FPS-R could possibly be applied as the parental pain observation tool as well. Consequently, we suggest that the parental assessment with the FLACC Scale and with the FPS-R might be more useful for detecting pain in young children with RTI, than the parental interview about their child’s pain, because pain scales might better freezeframe a moment for the parents to ponder their child’s pain, than the parental interview.

The occurrence of moderate/severe pain did not significantly differ between AOM and non-AOM groups with any of the three pain evaluation methods. At first sight, this may seem conflicting. However, symptoms of RTI may likewise cause severe distress to young children. In fact, when parents assessed their child’s pain with the FLACC Scale, which is validated to measure distress behavior, nasal congestion had a significant association with moderate/severe pain. Furthermore, ear-related pain may likewise accompany children with RTI due to the blocked ear and dysfunction of the Eustachian tube. Our current results also support our previous findings that symptoms of AOM and RTI are overlapping [6]. Our study illustrates the difficulties that the parents of young children are facing, when interpreting, which of the child’s symptoms are due to ear pain, or due to distress from RTI. Based on our preliminary results, we suggest that young children with RTI, without AOM, might suffer from equal amount of distress or discomfort as do children with AOM. Thus, when parents suspect their child with RTI to have AOM, we recommend that clinicians would actively offer pain medication, although AOM was not diagnosed. All in all, further studies are needed to investigate the severity of pain and its assessment in outpatient children with RTI.

The key symptoms associating with the parental assessment of their child to suffer from moderate/severe pain were ear pain reported by child and restless sleep. These two symptoms stood out, regardless of the pain evalution method. Indeed, restless sleep or fussiness has also previously been related as suggestive of ear pain in preverbal children [2, 4]. On the other hand, restless sleep has not been shown to resolve significantly faster with the antimicrobial treatment in children with AOM, compared to the treatment with the placebo [5]. Thus, this suggests that restless sleep may reflect the general pain and distress due to RTI, for example headache, sore throat or nasal congestion, rather than ear pain specifically. Interestingly, when pain was assessed by parents with the FPS-R, poor appetite seemed to be the sign for moderate/severe pain, although it has more commonly been held as a sign for child’s impaired overall condition. Hence, we suggest that if the validated pain scales are not available in the clinical practice, the clinician could ask about these specific symptoms (such as ear pain reported by child, restless sleep, poor appetite) to interpret whether a child with RTI suffers from moderate/severe pain.

Our study implies that the undertreatment of pain might be prevented in young children with the use of pain scales, such as the FPS-R and the FLACC Scale. This would have consequential impact on young children’s life, because pain experiences in early childhood may induce long-term alterations in pain sensitivity [32, 33]. Hence, our study might offer a valuable new perspective for clinicians who treat young children with RTI. Pain scales might be used as a simple tool at the primary care to explore the possible need for pain medication. However, more studies are mandatory before implementing pain scales for parental use in clinical practice.

Our study is not without limitations. First, due to the tight schedule at the study visit, we explained the pain scales to the parents very briefly, leaving parents a possibility of misunderstanding of matching the child’s facial expression to the faces in the FPS-R figure, despite the instructions. On the other hand, this reflects the real life in clinical practice and was thus also a strenght. However, it may be argued that the FPS-R is a relatively coarse scale with six categories for adult observers and that they would be capable of finer distinctions, for example with a finer-grained numerical rating scale. Second, to our experience, parents considered the FLACC Scale as challenging, because they had to recall their child’s behavior in each of the five behavioral categories, possibly causing recall bias. Third, the data about parental education level or occupation is missing, which may be seen as a limitation because higher level of parental education has been shown to be associated with higher reported pain levels [4]. However, our study population came from all the postal code regions of Turku area which shows the sosioeconomic heterogeneity of the population. Nonetheless, our study has also several strenghts. First, the standardized, structured symptom questionnaire allowed us to investigate the symptoms rigorously. Second, parents were surveyed about the symptoms via interview conducted by study physician. This represents well the actual real life situation in the primary care, reflecting generalisability of our results. Third, the diagnosis of AOM is firm due to our careful diagnostics [5]. This strenghtens our findings that children with RTI seem to suffer from moderate/severe pain, regardless of the diagnosis of AOM.

## Conclusions

The pain scales, such as the FPS-R and the FLACC Scale, might be more useful for parents to detect pain of young children with RTI, than the parental interview about pain. Equally important, the FPS-R and the FLACC Scale seem to indicate that the majority of children with RTI, either with or without AOM, might suffer from moderate/severe pain. Hence, we suggest that pain scales, such as the FPS-R and the FLACC Scale, might be used by parents in clinical practice. However, this is the first study to use the FPS-R and the FLACC Scale for parent observation in children with RTI. Hence, more studies are needed.

## Additional file

Additional file 1: Data set. (XLSX 46 kb)

## Abbreviations

AOM: Acute otitis media
CI: Confidence interval
FLACC Scale: Face, Legs, Activity, Cry, Consolability Scale
FPS-R: Faces Pain Scale-Revised
OR: Odds ratio
RTI: Respiratory tract infection
